# Guardianship and self-sovereign identity: implications for persons living with dessmentia

**DOI:** 10.1093/geroni/igab046.2667

**Published:** 2021-12-17

**Authors:** Noelannah Neubauer, Lili Liu

**Affiliations:** University of Waterloo, Waterloo, Ontario, Canada

## Abstract

Self-sovereign identity (SSI), an identity management system where individuals own and manage their digital identity, can improve access and management of one’s personal data. SSI is becoming feasible for the general public to use for their health and other personal data. Like any data system, when persons living with dementia no longer have capacity to provide informed consent, guardianship over their data is required. The purpose of this study was to examine the concept of guardianship within the context of SSI, specifically its application to persons living with dementia. This study followed a qualitative description approach. Seventeen semi-structured virtual interviews were conducted with persons living with dementia and care partners to elicit their perspectives on existing guardianship practices and guardianship within the context of SSI. Interviews were digitally recorded and transcribed verbatim. Conventional content analysis guided the analytic process. Participants had mixed impressions of existing guardianship practices. While some were positive, others thought existing practices failed to consider the complexity of caring for someone with dementia (e.g., presence of multiple guardians). Participants suggested that SSI has the potential to improve the security and safety of persons living with dementia who have had guardianship enacted (e.g., reduced risk of financial abuse.) Recommendations included ensuring that SSI guardianship processes are simple and flexible, building a user-friendly system that also considers the heterogeneity of persons living with dementia and their care partners. Overall, guardianship within the context of SSI was well received. Findings will be used to further inform the SSI guardianship processes.

